# Uncemented, curved, short endoprosthesis stem for distal femoral reconstruction: early follow-up outcomes

**DOI:** 10.1186/s12957-018-1486-3

**Published:** 2018-09-10

**Authors:** Minxun Lu, Jie Wang, Cong Xiao, Fan Tang, Li Min, Yong Zhou, Wenli Zhang, Chongqi Tu

**Affiliations:** 1 Department of Orthopedics, West China Hospital, Sichuan University, No. 37 Guoxue Street, Chengdu, 610041 People’s Republic of China; 2 Department of Orthopedics, The Third Hospital of Mianyang, No. 190 The East Jiannan Road, Mianyang, 621000 Sichuan People’s Republic of China

**Keywords:** Uncemented, Curvature, Distal femur, Stem, Short

## Abstract

**Background:**

Uncemented endoprosthetic knee replacement has become a mainstream treatment for malignant tumours of the distal femur. Most femoral stems, however, are straight and therefore poorly fit the anteriorly bowed curvature of the femur. To address this issue, we used a short, curved, uncemented press-fit femoral stem and evaluated its short-term outcomes after reconstruction of the distal femur.

**Methods:**

Forty-two patients underwent distal femur replacement using curved press-fit stem. To assess the interface, we measured the axial length of the press-fit area and the perpendicular distance of the radiolucent area between the stem and bone on digital images obtained using tomosynthesis with Shimadzu Metal Artefact Reduction Technology (T-SMART). Postoperative complications and oncological outcomes were monitored at each follow-up visit.

**Results:**

Of the 42 patients enrolled in the study, two had cancer-related deaths and one had local tumour recurrence. The minimum follow-up time of the surviving patients was 24 months, with no incidence of aseptic loosening or mechanical failure of the prosthesis. The average effective contact length between the press-fit stem and bone was 74.0 mm, with nearly undetectable radiolucent gaps between the implant and the bone on medial-lateral and anteroposterior views.

**Conclusions:**

Over the short term, uncemented, curved, short stem provides a stable bone-prosthesis interface without any aseptic loosening.

## Background

Primary musculoskeletal tumours are common in the distal femur. The introduction of neo-adjuvant chemotherapy and improvement in surgical techniques and prosthesis designs have allowed an endoprosthetic replacement to become the standard method of reconstruction and limb salvage after resection of distal femoral tumours [1, 2]. However, various complications of endoprosthetic replacement are frequently encountered, including aseptic loosening, mechanical failure, infection, and periprosthetic fracture [3]. The rate of these complications is influenced by the type of fixation and the design of the prosthesis, especially for the femoral stem.

The press-fit fixation has been associated with a lower rate of aseptic loosening than cemented fixation and thus is a more reasonable method of stem fixation. However, providing adequate primary stability is a necessity for press-fit fixation. Currently, the Global Modular Replacement System [4] (GMRS, Stryker Orthopaedics, Mahwah, USA), the Kotz Modular Femur and Tibia Reconstruction System [5] (KMFTR, Howmedica GmbH, Kiel, Germany), the Modular Universal Tumour and Revision System [6] (MUTARS, Implantcast GmbH, Buxtehude, Germany), the Segmental System [7] (Zimmer Inc., Warsaw, IN, USA), and the Megasystem-C [7] (LINK GmbH, Hamburg, Germany) are acceptable choices for distal femoral reconstruction (DFR). Although these various designs do provide adequate mechanical strength and primary stability, these commercially available uncemented stems use a straight femoral stem, except the MUTARS and Segmental System. Obviously, a straight stem design is a mismatch for the anterior curvature of the medullary cavity of the femur. The MUTARS and Segmental System do provide a curved stem; its match to the anatomical femoral curvature of Chinese patients is not clear. We propose that short, curved, uncemented femoral stems with derotational fins would be a better design for femoral stems used in DFR. To our knowledge, however, the clinical outcomes of these stems for DFR implants have not previously been evaluated. Therefore, our aim in this study was to retrospectively evaluate the short-term outcomes of using short, curved, uncemented femoral stems for DFR, either after tumour resection or for DFR revision.

## Methods

### Ethical considerations

This study was approved by the Institutional Review Board and performed in accordance with the ethical principles of the Declaration of Helsinki. All patients provided written informed consent.

### Patients

Between October 2014 and December 2017, 42 patients underwent DFR with uncemented, curved, short stems after resection of malignant tumours or for DFR revision in our Department of Orthopaedics. The study group included 25 men and 17 women, with a mean age of 29.3 years (range, 10–67 years). Among these cases, 35 were primary implantation procedures and 7 were revisions necessary due to aseptic loosening of the cemented prosthesis. Pre-operatively, the length of required resection, the percentage of resected bone required, and the radius of curvature (ROC) of the retained femur were measured on plain radiographs. Follow-up assessments were performed every 3 months, postoperatively, during the first year and at every 6 months, thereafter. Lower limb function, the condition of the bone-prosthesis interface, the presence of complications, and oncological outcomes were assessed at each follow-up visit. Lower limb function was evaluated using the Musculoskeletal Tumor Society (MSTS) scoring system [8]. The condition of the bone-prosthesis interface was assessed using digital T-SMART and plain radiography, measuring the axial length of the press-fit area and radiographic evidence of bone resorption. The axial press-fit length was defined as the distance from the distal point of the stem to the location where the perpendicular distance between the stem and the periprosthetic bone was < 1 mm, and the final axial length was calculated by averaging the axial press-fit length in the medial-lateral and anterior-posterior planes. The periprosthetic radiolucent area, which indicates bone resorption, was evaluated at six points on the femoral stem, namely, the distal endpoint, midpoint, and proximal endpoint measured on bone anterior-posterior and medial-lateral radiographic views. We also evaluated radiographic variables of bone ingrowth into the prosthesis, including bone bridging, spot welding, and neocortex formation. Complications related to stem implantation, including infection, aseptic loosening, periprosthetic fracture, and breakage, were evaluated. All data were analysed using SPSS, version 19, software.

### Stem design

**Fig. 1:**
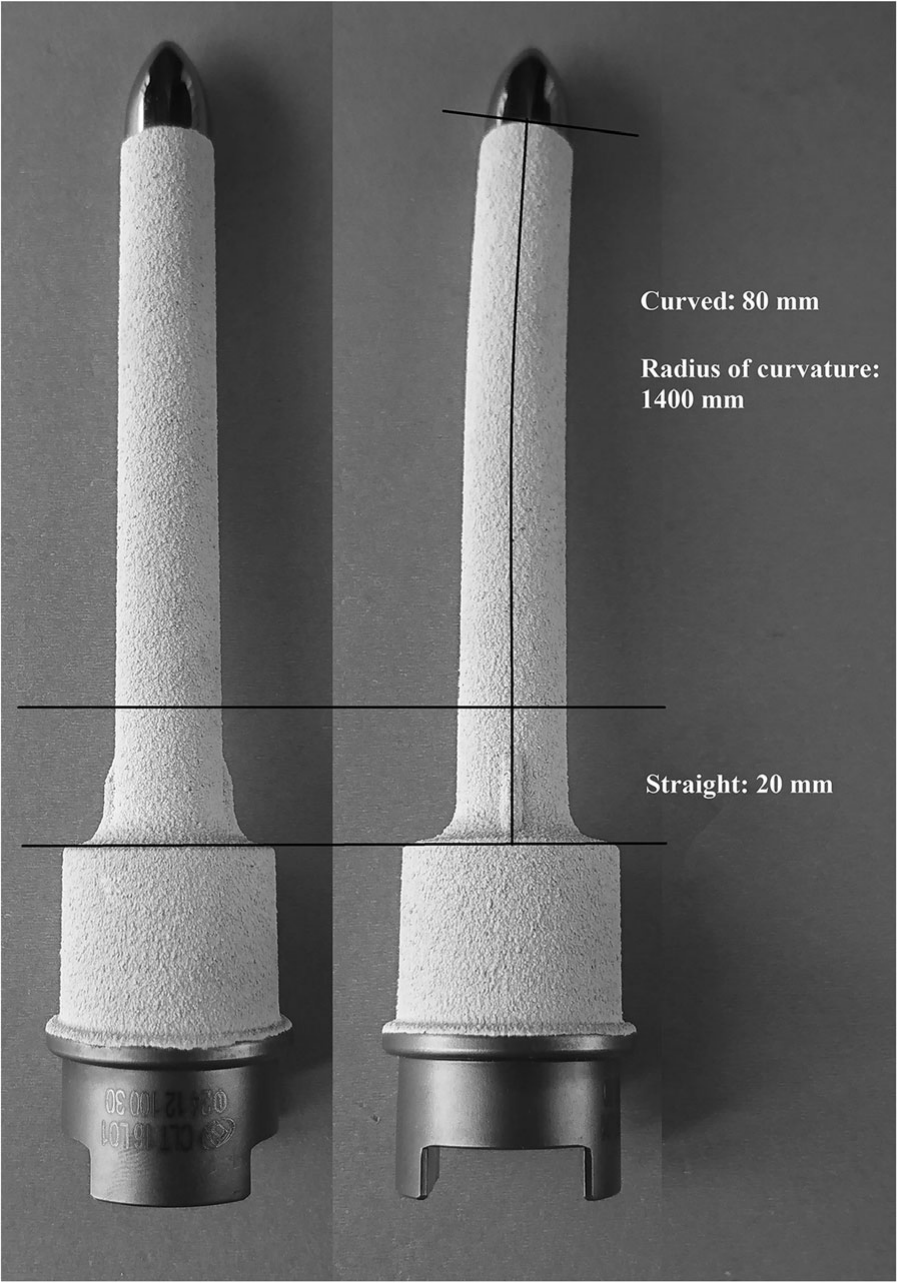
Design specification and schematic cross sections of the curved femoral stem

All of the curved stems were fabricated by Chunlizhengda Medical Instruments (Tongzhou, Beijing, China). Of the 42 stems that were implanted, 7 were custom-made for DFR revision and 35 were standard modular stems for regular DFR. The standard stem had various diameters, ranging between 10 and 18 mm, and a length of 100 mm. The distal part of the stem was 20 mm in length and straight, with two symmetrically arranged fins (medial and lateral), with the rest of the stem having an anterior curvature (radius, 1400 mm) to match the shape of the medullary canal of the femur (Fig. 1). The stem was coated with hydroxyapatite (HA). The custom-made stems used the same standard design but were longer and/or larger.

### Surgical technique

**Fig. 2:**
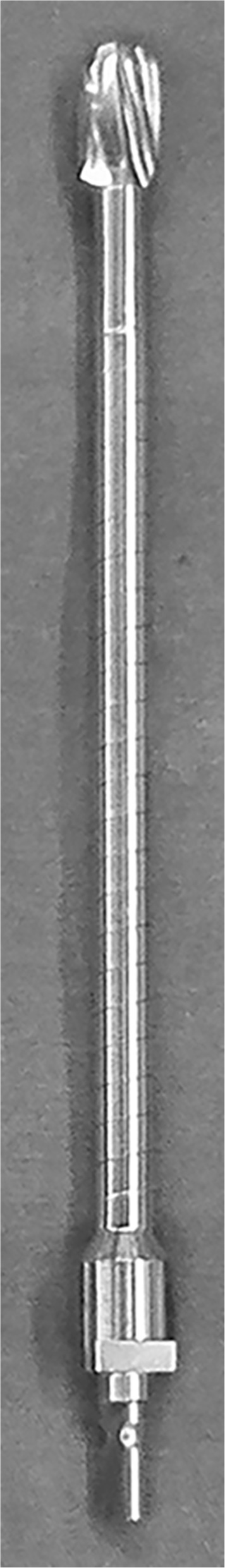
The flexible reamer we used

All surgeries were performed by a senior surgeon (C. Tu). The degree of tilt of the osteotomy plane was precisely controlled to minimize the potential for a misfit between the standard stem and the ROC of the femur. After segmental resection of the tumour, we used a flexible reamer, with a guide line in the centre, to enlarge the medullary cavity of the femur, maintaining its anterior curvature (Fig. 2). To minimize bone loss and maximize primary stability of the endoprosthesis, the femoral canal was under-reamed by 0.5 mm, compared to the diameter of the stem, with subsequent adjustments, in 0.5-mm increments, as needed, until a stable press-fit was achieved. After completing the reaming process, we drilled the medial and lateral tracts, using mini drill bits to minimize the risk of fracture, to allow the fins to be inserted. When implanting the stem, the rotation was easily controlled by using the position of the fins as a guide.

### Postoperative management

Partial weight-bearing, using two crutches, was initiated on postoperative day 3, with active range of motion exercises of the knee also initiated on that day. Progression to full weight-bearing, using two crutches, was initiated on postoperative week 2.

## Results

**Fig. 3:**
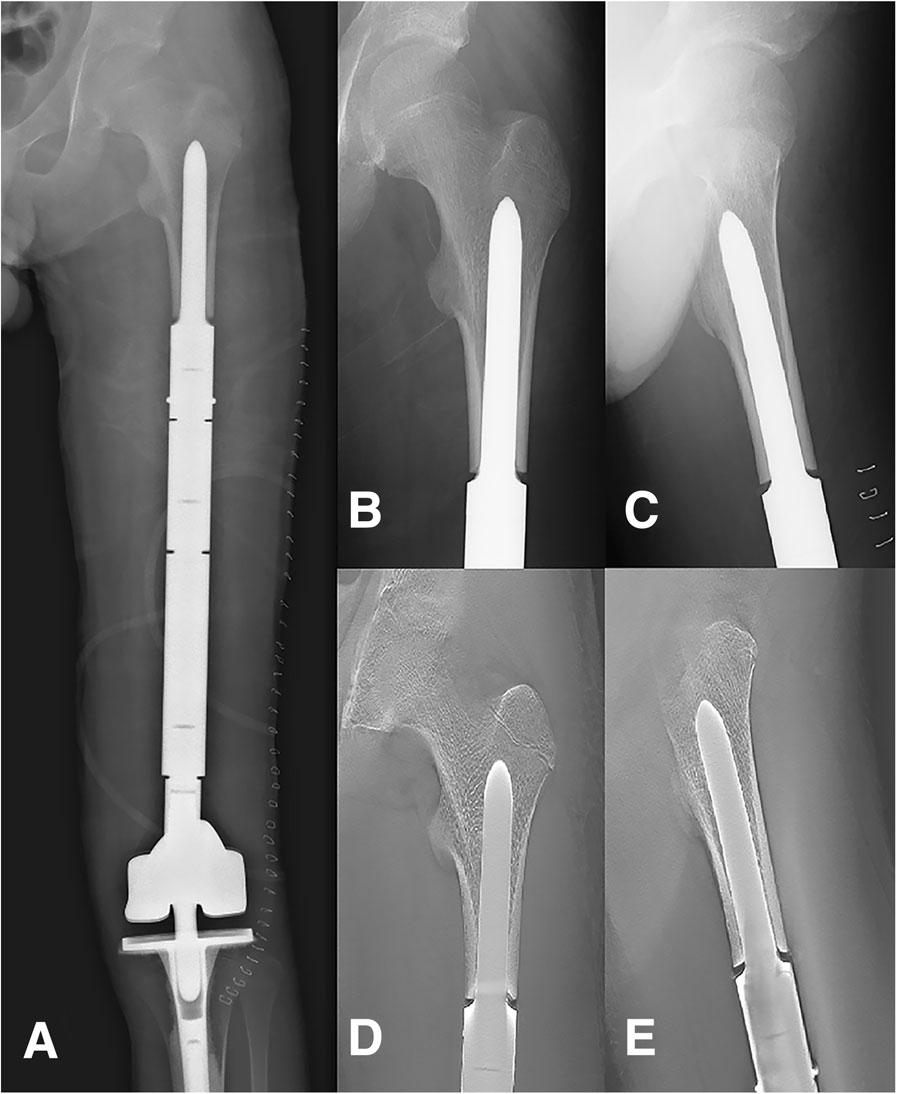
A case of reconstruction of the distal femur following distal resection of 73% of the length of the femur. **a** Postero-anterior radiograph of the entire femur. **b**, **c** Postero-anterior and lateral radiographic views of the region of stem insertion of the femur. **d**, **e** Postero-anterior and lateral T-SMART views of the stem insertion region of the femur

**Fig. 4:**
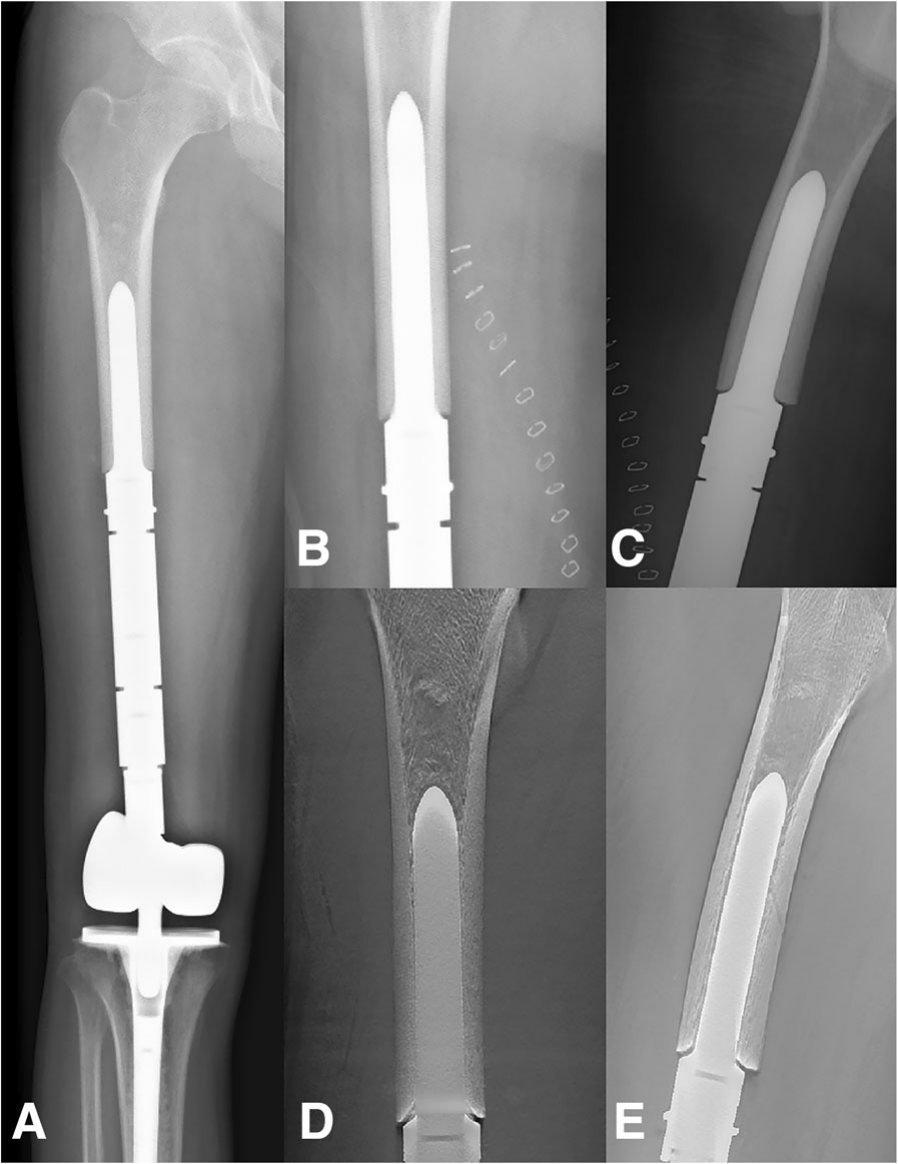
A case of reconstruction of the distal femur following a distal resection of 54% of the length of the femur. **a** Postero-anterior radiograph of the entire femur. **b**, **c** Postero-anterior and lateral radiographic views of the region of stem insertion of the femur. **d**, **e** Postero-anterior and lateral T-SMART views of the stem insertion region of the femur

**Fig. 5:**
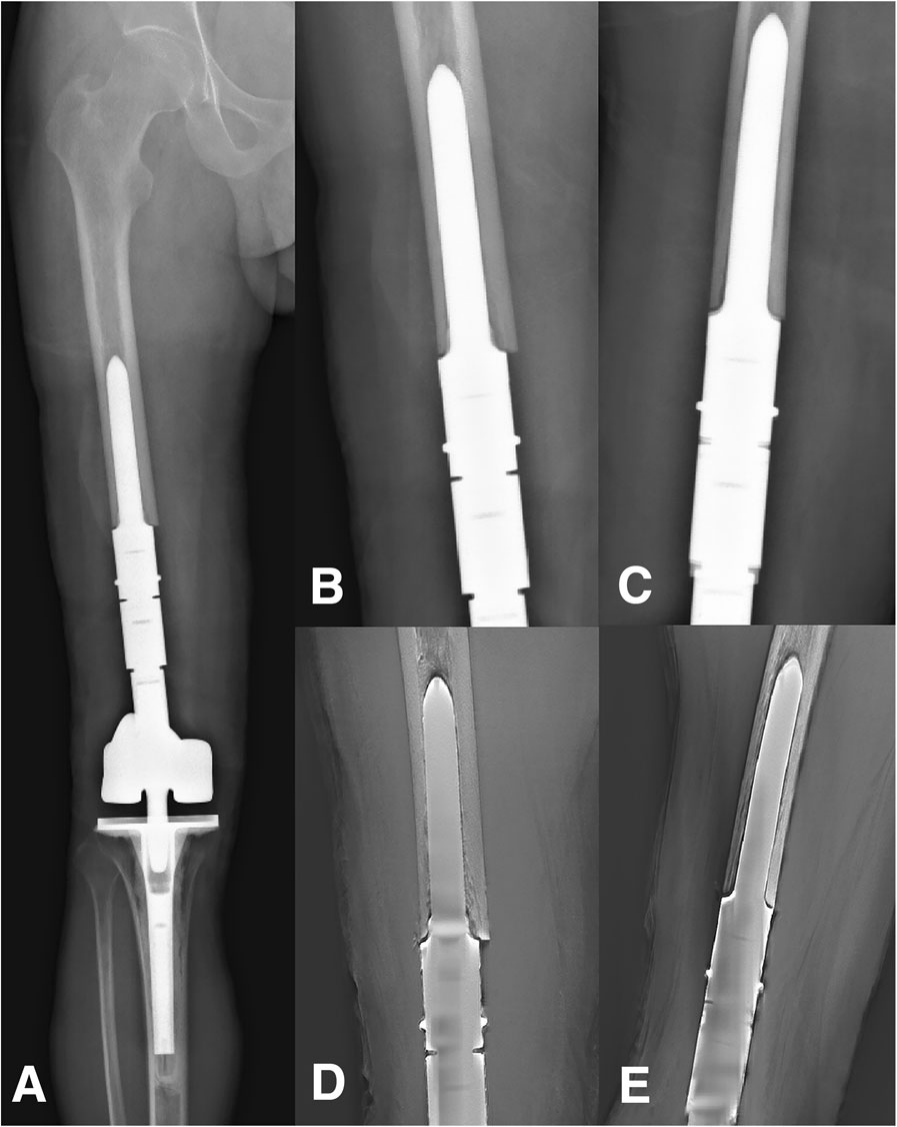
A case of reconstruction of the distal femur following a distal resection of 26% of the length of the femur. **a** Postero-anterior radiograph of the entire femur. **b**, **c** Postero-anterior and lateral radiographic views of the region of stem insertion of the femur. **d**, **e** Postero-anterior and lateral T-SMART views of the stem insertion region of the femur

Of the 42 patients enrolled in the study, two died of lung metastases (average survival time, 16.5 months). The average follow-up duration for the remaining 40 patients was 30.1 months (range, 24–41 months). Three stems were inserted into the proximal femur after resection of > 60% of the length of the femur due to massive tumour resection (Fig. 3), with another 14 stems inserted into the mid-section of the femur after resection of 40–60% of the length of the femur (Fig. 4). For the remaining 23 cases, less than 40% of the length of the femur was resected (Fig. 5). The length of the resected femur ranged between 61.1 and 313.7 mm (average, 157.9 mm), with an average radius of 1347.5 mm retained (range, 820–1620 mm). Among the 40 patients who survived the period of observation, local recurrence occurred in one patient. With regard to lower limb function, the average MSTS score was 85.8 ± 7.45%. Periprosthetic infection was the only complication observed, developing in three cases (7.5%; Table 1), with all three being primary implantation cases (Table 2). Of these three cases, two were treated with debridement, drainage, and antibiotics, without removal of the prosthesis. In the other case, amputation was required. There was no incidence of implant fractures, mechanical failure, or aseptic loosening.

**Table 1:** Oncology outcomes and complications related to the percentage of bone resection

Group	Number of patients	Age, years	Primary/revision	Length of resection, mm	Percentage of bone resection, %	Radius of retained femur, mm	Oncology outcome	Amputation	Complications		
									Loosening, %	Breakage, %	Infection
< 40% resection	23	33.8 (range, 14–67)	16/7	118.7 (range, 61.1–164.2)	29.8 (range, 15–39)	1470 (range, 1230–1620)	0%	4.3% (1/23)	0	0	8.7% (2/23)
40 to 60% resection	14	22.5 (range, 10–62)	14/0	188.7 (range, 160.5–223.9)	49.2 (range, 41–58)	1210 (range, 1010–1430)	Local recurrence 7.1% (1/14)	0%	0	0	7.1% (1/14)
> 60% resection	3	26.3 (range, 14–50)	3/0	301.3 (range, 294.0–313.7)	73.4 (range, 69–78)	1050 (range, 820–1170)	0%	0%	0	0	0%
All	40	29.3 (range, 10–67)	33/7	157.9 (range, 61.1–313.7)	40.2 (range, 15–78)	1347.5 (range, 820–1620)	2.5% (1/40)	2.5% (1/40)	0	0	7.5% (3/40)

**Table 2:** Oncology outcomes and complications related to the type of implant fixation

Type of implant	Number of patients	Age/years	Oncology outcome	Amputation	Complications		
					Loosening, %	Breakage, %	Infection
Primary	33	29.0 (range, 10–67)	Local recurrence 3% (1/33)	3% (1/33)	0	0	9.1% (3/33)
Revision	7	30.7 (range, 21–45)	0%	0%	0	0	0%
All	40	29.3 (range, 10–67)	2.5% (1/40)	2.5% (1/40)	0	0	7.5% (3/40)

**Fig. 6:**
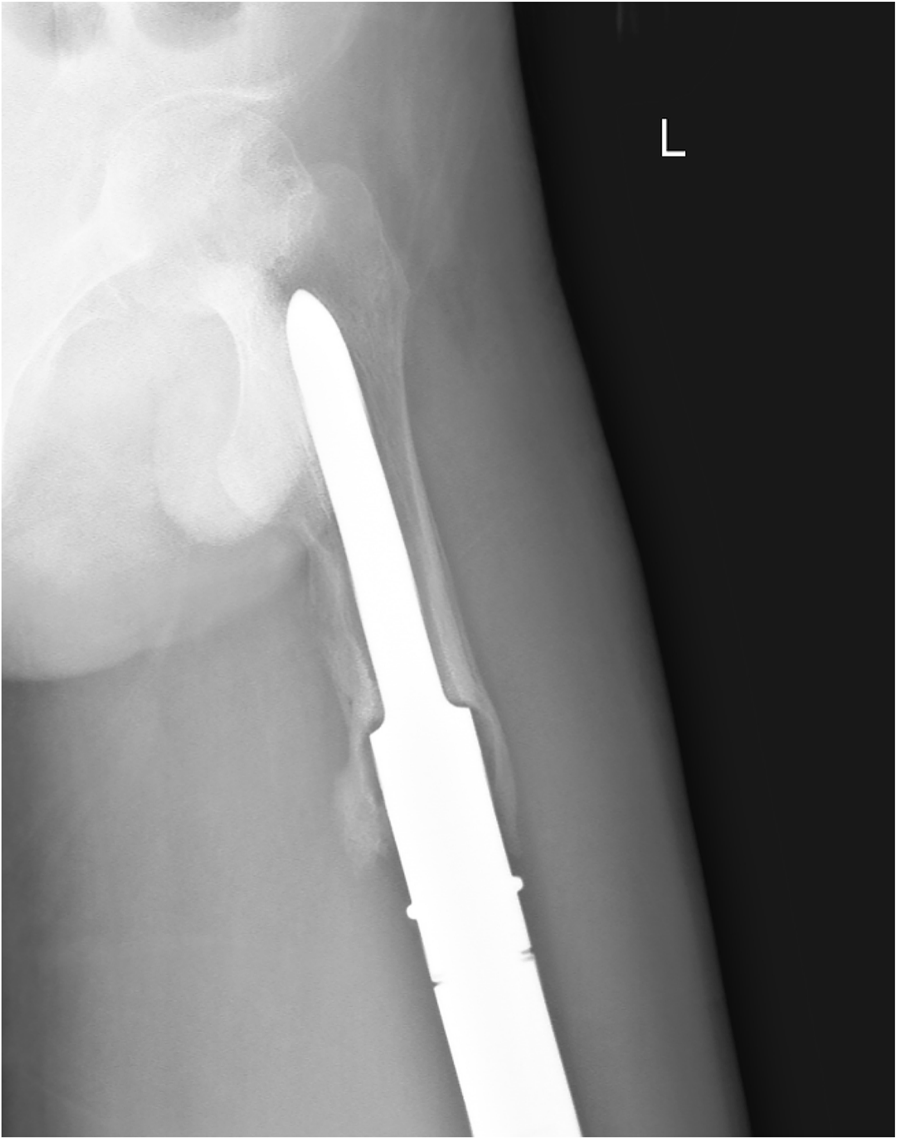
A typical case of bone ingrowth is shown

The average effective contact length between the press-fit stem and bone was 74.0 mm. The mean gaps between the bone and the stem in the medial and lateral planes, respectively, were as follows: distal endpoint of the stem, 0.24 and 0.16 mm; midpoint, 0.98 and 0.53 mm; and proximal endpoint, 1.09 and 0.86 mm. The mean gaps in the anterior and posterior planes, respectively, were as follows: distal endpoint of the stem, 0.61 and 0.65 mm; midpoint, 1.03 and 0.92 mm; and proximal endpoint 1.26 and 1.28 mm (Table 3). Radiographic signs of bone ingrowth were identified in all stems, with the exception of the patient who developed a periprosthetic infection requiring amputation and the patient with local tumour recurrence. A typical case of postoperative neocortex formation is shown in Fig. 6.

**Table 3:** Bone-implant interface evaluation related to the percentage of bone resection

Group	Number of patients	Length of retained femur, mm	Axial length of press-fit area, mm	Percentage of press-fit length in stem length, %	Vertical distance of radiolucent area					
					Anterior/posterior			Medial/lateral		
					Distal endpoint, mm	Midpoint, mm	Proximal endpoint, mm	Distal endpoint, mm	Midpoint, mm	Proximal endpoint, mm
< 40% resection	23	279.8 (range, 237.7–353.6)	92.3 (range, 80.7–136.6)	91.8 (range, 80.1–97.5)	0.52/0.61	0.92/0.75	1.12/0.90	0.21/0.17	0.45/0.34	1.02/0.83
40 to 60% resection	14	195.0 (range, 150.4–246.4)	57.0 (range, 21.7–87.1)	57.0 (range, 21.7–87.1)	0.78/0.84	1.08/1.03	1.55/2.1	0.35/0.18	0.64/0.45	1.24/0.94
> 60% resection	3	110.0 (range, 83.2–131.4)	20.5 (range, 15.8–24.3)	20.5 (range, 15.8–24.3)	0.63/0.3	2/2.2	N/A	0/0	2.1/2.3	N/A
All	40	236.3 (range, 83.2–353.6)	74.0 (range,15.8–136.6)	74.3 (range, 15.8–97.5)	0.61/0.65	1.03/0.92	1.26/1.28	0.24/0.16	0.98/0.53	1.09/0.86

*N/A* not applicable

## Discussion

Many types of cemented [3, 9–14] and uncemented [4–6, 15–19] prostheses have been used for DFR after tumour resection (Table 4). Although the optimal method of endoprosthesis fixation in the host bone is still controversial, uncemented stems are now more commonly selected than cemented stems because of the advantages they provide. Foremost, press-fit fixation of uncemented stems can easily be achieved without the need for highly demanding bone cementation. Moreover, uncemented stems with HA coating facilitate biological bone ingrowth at the bone-prosthesis junction, as well as have a lower rate of complications [4–6, 15, 16, 18–20] than cemented stems. In the previous study, Biau et al. [9] followed up 56 patients who had undergone cemented, custom-made megaprosthesis reconstruction, between 1972 and 1994, for the treatment of distal femoral tumours. After an average follow-up of 62 months, limb function was acceptable but with femoral stem loosening reported in eight cases and stem fracture in five. In 2007, Myers et al. [12] reported on the long-term outcomes of cemented endoprosthetic replacement of the distal femur after tumours resection. Between 1973 and 2000, 335 patients underwent DFR. After a mean follow-up period of 12 years, the risk of revision for aseptic loosening of a fixed hinge was nearly 35% at 10 years, compared to 24% for a rotating hinge. Compared to cemented fixation, O’Donnell et al. [4] evaluated the early results of a custom-made, non-fluted, press-fit GMRS stem in 35 patients who had undergone DFR. Aseptic loosening and stem fracture were not observed during the minimum follow up period of 22 months. Additionally, a low rate of aseptic loosening or fracture in uncemented KMFTRS stems was reported by Griffin et al. [5], with femoral loosening identified in two cases (2.7%) and fracture in four (5.4%), between 1989 and 2000. Furthermore, in their 117 patients who underwent uncemented modular tumour prosthetic reconstruction (using the KMFTRS and MUTARS systems) for distal femoral tumours, Song et al. [16] reported that prosthesis removal was required in 35 cases (30%), 17 (14.5%) of these due to infection, 5 (4.2%) due to local tumour recurrence, 7 (5.9%) due to stem loosening, 4 (3.4%) due to stem fracture, and 2 (1.2%) due to periprosthetic fracture, over an average follow-up of 95 months. Our short-term outcomes on using a short, curved, femoral stem were comparable to those of previous studies using uncemented stems. The average MSTS score was good overall, with postoperative lower limb function not being a limitation to activities of daily living. Knee function was restored to a satisfactory level, even in patients who underwent massive bone resection (> 60% of the length of the femur). Over our average short-term follow-up of 30.1 months, we identified three cases of periprosthetic infection, with no radiographic evidence of aseptic loosening or breakage. As such, periprosthetic infection was the most common complication in our study group. The previous studies reported an infection rate for primary endoprostheses of 2–20% [9, 21–23], increasing to 43% after revision surgery [24]. But, inconsistently, Pala et al. [17] reported a lower rate of infection in their case series of 98 patients who underwent revision surgery, over a mean oncologic follow-up of 4.2 years (range, 2–8 years), with infection identified in 7 cases, compared to 21 cases of infection in 197 cases of primary implantation. The findings reported by Pala et al. are comparable to ours, with no cases of infection identified in the revision group, and three in the primary implantation group. The potential risk factors of infection for oncologic patients are insufficient soft tissue coverage, immune compromising treatments, length of the procedure, and extensive surgical dissections [22, 23, 25]. Although the mean follow-up of 31 months was not sufficiently long to directly validate the rate of prosthesis-related complications, loosening is not likely to occur after bony ingrowth of a HA-coated implant has taken place for biological reconstruction [26]. However, the time to sufficient bone ingrowth, which produces a relatively high pullout force, is approximately 12 months [27]. Thus, the possibility of apparent loosening is lower in the second year after surgery than in the first year. In Pala et al.’s study [17], which focused on uncemented types of fixation for DFR, loosening contributed to endoprosthesis failure in 15 cases, requiring 2.8 years to develop, on average. Similar results were also reported by Bus et al. [6], with 15 cases of aseptic loosening identified in 89 cases of DFR performed using a MUTARS endoprosthesis, with a median time of 1.2 years. Biomechanically, it is true that the relatively high level of force would be applied on the interface between the stem and bone in the very early postoperative period for DFR because of a long lever arm of endoprostheses [4]. With insufficient bone ingrowth interface during this period, loosening or failure would be more likely to take place during this period when osteointegration is occurring rather than in the period after achieving a well-integrated interface. Thus, we believe that the majority of loosening cases would be readily apparent even at this early time point of follow-up.

**Table 4:** Previous studies for distal femur reconstruction

	Time span	Prosthesis type	Major fixation type	Number of patients*	Loosening, %	Implant fracture, %	5-year survival, %
Unwin et al. [3]	1968–1992	Custom-Stanmore	Cemented	493	9.9	3	N/A
Myers et al. [12]	1973–2000	Custom-Stanmore	Cemented	335	N/A	2	N/A
Schwartz et al. [13]	1980–2008	Custom or modular	Cemented	186	11.8	5.3	87.7
Frink et al. [10]	1983–1999	Stryker	Cemented	74	9.4	2.7	N/A
Jeys et al. [11]	1986–1996	Custom	Cemented	228	13.6	2.2	N/A
Griffin et al. [5]	1989–2000	KMFTR	Uncemented	74	2.7	5.4	N/A
Wunder et al. [15]	1986–1995	KMFTR	Uncemented	50	2	8	90
Song et al. [16]	1988–2008	KMFTR MUTARS	Uncemented	117	5.9	3.4	74
Batta et al. [19]	1994–2006	Custom	Uncemented	69	13	10	72.7
Bus et al. [6]	1995–2010	MUTARS	Uncemented	89	17	N/A	N/A
Pala et al. [17]	2003–2010	GMRS	Uncemented	187	5.3	0	N/A
O’Donnell et al. [4]	2005–2012	GMRS	Uncemented	35	0	0	N/A
Current study	2015–2017	Curved stem	Uncemented	42	0	0	N/A

*GMRS* Global Modular Replacement System, *KMFTR* Kotz Modular Femur and Tibia Reconstruction System, *MUTARS* Modular Universal Tumour and Revision System, *N/A* not applicable

*Number of patients who underwent distal femoral replacement

Another strength of our study is our precise measurement of the radiolucent area between the periprosthetic bone and the implant, indicating potential bone resorption which may be a more prevalent complication in uncemented implants over longer follow-up periods [3, 18, 28]. We observed a slightly larger gap between the bone and the prosthesis in the anterior-posterior plane than in the medial-lateral plane, with the largest gap being 2 mm in length, which is considered a relatively slight resorption of the periprosthetic bone and unlikely to lead to loosening or breakage. Furthermore, there was minimal gapping at the distal endpoint and mid-section of the stem in either the medial-lateral or anterior-posterior plane, indicating that these sections of the interface were well integrated and effectively constrained the movement of the stem. It is true that having a minimal tiny gap is of beneficial to generate friction between the stem and the bone, this friction acting as a shear stress and resulting in a certain degree of stress shielding. But, considering that the interface between the stem and the bone is the first and major location bearing physical force in DFR, a minimal gap (or no gap at all) was required for press-fit fixation to produce and maintain sufficient pullout and rotation stability. Although the biomechanical outcomes related to DFR have yet to be fully characterized, we believe friction and compression to be the primary modes of load transfer between the stem and bone once bone ingrowth into the interface has developed. By contrast, during the very early postoperative period, the compressive stress placed on the collar of the stem is the main force, due to the immature formation of the bone ingrowth at the interface and a slightly larger gap caused by the drilling of a sufficient canal to insert a curved stem.

In addition to the anatomically appropriate curvature of the stem, the suitable length of the prosthesis, with sufficiently strong mechanical properties, two anti-rotational fins, and HA coating of the stem, provide a good functional outcome with a low risk of complications.

After implantation of a curved stem, strong derotational forces would be naturally generated because of space restriction. Such restraint is not available when using a straight stem as the space required for implantation is exactly the same as that allowing rotation of the stem. The curved stem, particularly with a rough surface, provides a larger contact surface between the prosthesis and bone than a straight stem with same parameters, which generates greater friction to enhance primary pull-out and rotational stability. Lastly, the curved stem avoids unnecessary destruction of cortical bone at the proximal endpoint of the stem. Currently, the GMRS, KMFTR, MUTARS, Segmental System, and Megasystem-C are frequently used distal femoral endoprosthetic reconstruction systems with uncemented stems (Table 5). Although the MUTARS and Zimmer systems provide a curved stem, the details of the stem design are not open-source. Considering the major markets for both systems, the design of the stem is more likely to be appropriate for European and/or American populations than for Asians. A significant difference in femoral bowing between different ethnicities was reported by Maratt et al. [29]. Some studies reported that the average ROC of the femoral canal for Chinese patients ranged approximately from 1100 [30] to 1500 mm [31]. Thus, the ROC of stem we used (1400 mm) might be more suitable for the Chinese patients. According to the data related to femoral bowing we collected, there is a huge variation in the radius of the femoral curvature, ranging between 820 and 1620 mm. For all cases, we used a stem with a constant radius of 1400 mm rather than using personalized custom-made or modular stems with various radii. Although, customized and modular stems with different radius would provide a closer matching rate between the curvature of the stem and the femoral canal, designing and fabricating a customized stem requires 2 to 3 weeks, which could result in tumour progression and metastasis, while the use of modular stem with a range of radii would unnecessarily increase the cost of stems. Furthermore, we believe that any mismatch between a stem with a fixed radius of 1400 mm could be minimized through adjustment in the degree of prosthesis tilt, even in patients having the smallest femoral curvature of 820 mm.

**Table 5:** Design features of common commercially available uncemented stems for distal femur reconstruction

Implant		GMRS Stryker	MUTARS Implantcast	Megasystem-C LINK	Segmental system Zimmer	Current study Chunli Co.
Alloy		TiAl_6_V_4_	TiAl_6_V_4_	TiAl_6_V_4_	CoCrMo	TiAl_6_V_4_
Design	Global	Straight Cylindrical	Curved Hexagonal	Straight Cylindrical	Straight/curved Conical	Partially Curved Cylindrical
	Proximal	Four fins	–	Fluted	Trabecular metal collar	Straight with two fins
	Middle	–	–	Fluted	Fluted	–
	Distal	Tapered	Cylindrical	Fluted	Fluted Double-slotted	Tapered
Radius of curvature, mm		–	–	–	–	1400
Diameter, mm		8–17	12–18	12–24	9–19	10–18
Length, mm		105–325	120/160/200	100/130/160	130/190	100/110/120
Surface	Proximal	HA-coated/porous-coated	HA-coated	Porous-coated	–	HA-coated
	Middle	Porous-coated	HA-coated	Porous-coated	–	HA-coated
	Distal	–	Polished	Porous-coated	–	Polished

*GMRS* Global Modular Replacement System, *MUTARS* Modular Universal Tumor and Revision System, *HA* hydroxyapatite

A distinct advantage of using a short, rather than long, stem for DFR is the reduced volume of bone loss which results from the over-reaming procedure for longer stem implantation. The short stem also increases the indications for limb salvage by allowing prosthesis implantation in cases with a relatively short residual proximal femur due to extensive resection. Moreover, a short stem could decrease the stress shielding effect, which is a major reason for bone resorption and even aseptic loosening. As well, Levadnyi et al. [32] reported that long stems do not effectively transmit load to bone, whereas a short stem provides a favourable environment for load transfer to the proximal region, which allows bone density to be maintained. Furthermore, the mechanical strength of a short stem is similar to that of a long stem. Zdero et al. [33] tested four different stems, including the Sigma Short Stem, Sigma Long Stem, Genesis II Short Stem, and Genesis II Long Stem, to evaluate their mechanical properties, reporting that there was no significant difference between the short and long stems in terms of axial, lateral, and torsional stiffness. Lastly, it is important to note that the insertion procedure for a short stem is much easier and quicker than for a long stem.

The short, curved stem we used has two fins, symmetrically arranged in the true medial and lateral planes of the distal end of the stem, providing guidance for implantation and an additional derotational force. Although some surgeons [4] have suggested that channels drilled into the cortical bone to allow for the insertion of fins might increase the risk of bone fracture, the fins in the stem we used are not large enough to result in such complication.

During surgical implantation, adjustment of the osteotomy plane is one of the most important components of the procedure to minimize the effect of mismatching between the standard stem and femoral bowing before insertion. As an example, when the radius of the retained femur is smaller than the radius of the stem, slightly more cortical bone could be removed from the anterior than posterior aspect, such that the stem would be in a slight posterior tilt and, thus, decreasing the degree of a mismatch after implantation. Similarly, the stem could be positioned in an anteverted position by resecting a greater proportion of the bone on the posterior than anterior aspect in cases in which the radius of the retained femur is larger than the radius of the stem. For press-fit implantation of the curved stem, the reaming process is another essential component of the surgical procedure. Currently, the most effective ratio between the diameter of the press-fit stem and the diameter of the reamed canal is controversial. Although some investigators have suggested that the medullary cavity should be either under-reamed by 1 to 1.5 mm or reamed to exactly match the diameter of the stem [6, 34], in our clinical experience, we have found that the canal should be reamed to be 0.5 to 1 mm larger than the diameter of the curved stem to be inserted.

This study has some limitations that should be acknowledged. The duration of follow-up was not sufficiently long to verify the long-term efficacy of this uncemented, short, curved stem we used. However, considering the relatively strong forces on the stem-bone interface because of the long lever arm of the prosthesis and lack of well-formed bone ingrowth in the very early period after the surgery, aseptic loosening or failure of the osseointegrated interface would be more likely to occur in the very early period of follow-up. Although there is a possibility that more complications might arise as we follow these patients over a longer period of time, we believe that our clinical and radiographic assessments would be helpful to estimate the long-term survival of implants. As musculoskeletal tumours are relatively rare, our study sample was small and we did not have a control group. Therefore, a larger multicentre study is needed to compare this approach with other types of stems.

## Conclusions

Reconstruction using an uncemented, curved, short stem can be an alternative treatment option DFR after resection of the primary bone or metastatic tumours of the distal femur. On the basis of our results, we suggest that selection of a short, curved stem; careful reaming and insertion without any rotation; and press-fit fixation lead to reasonable postoperative knee function and a low risk of complications.

## Abbreviations

DFR: Distal femoral reconstruction
GMRS: Global Modular Replacement System
HA: Hydroxyapatite
KMFTR: Kotz Modular Femur and Tibia Reconstruction system
MSTS: Musculoskeletal Tumor Society
MUTARS: Modular Universal Tumour and Revision System
OSS: Orthopaedic Salvage System
ROC: Radius of curvature
T-SMART: Tomosynthesis with Shimadzu Metal Artefact Reduction Technology
